# Advancements in Chitosan-Based Nanoparticles for Pulmonary Drug Delivery

**DOI:** 10.3390/polym15183849

**Published:** 2023-09-21

**Authors:** Thiago Medeiros Zacaron, Mariana Leite Simões e Silva, Mirsiane Pascoal Costa, Dominique Mesquita e Silva, Allana Carvalho Silva, Ana Carolina Morais Apolônio, Rodrigo Luiz Fabri, Frederico Pittella, Helvécio Vinícius Antunes Rocha, Guilherme Diniz Tavares

**Affiliations:** 1 Postgraduate Program in Pharmaceutical Science, Federal University of Juiz de Fora, Juiz de Fora 36036-900, Minas Gerais, Brazil; t.zacaron@gmail.com (T.M.Z.); mirsih.pc@gmail.com (M.P.C.); dominiquefarmacia@gmail.com (D.M.e.S.); allanacarvalho23009@gmail.com (A.C.S.); rodrigo.fabri@ufjf.br (R.L.F.); frederico.pittella@ufjf.br (F.P.); 2 Faculty of Pharmacy, Federal University of Juiz de Fora, Juiz de Fora 36036-900, Minas Gerais, Brazil; mariana.leite@estudante.ufjf.br; 3 Postgraduate Program in Dentistry, Federal University of Juiz de Fora, Juiz de Fora 36036-900, Minas Gerais, Brazil; carolinaapolonio@gmail.com; 4 Laboratory of Micro and Nanotechnology—Farmanguinhos, FIOCRUZ—Fundação Oswaldo Cruz, Rio de Janeiro 21040-361, Rio de Janeiro, Brazil; helvecio.rocha@fiocruz.br

**Keywords:** lung diseases, pulmonary drug delivery, lung delivery, chitosan, chitosan derivatives, nanoparticles

## Abstract

The evolution of respiratory diseases represents a considerable public health challenge, as they are among the leading causes of death worldwide. In this sense, in addition to the high prevalence of diseases such as asthma, chronic obstructive pulmonary disease, pneumonia, cystic fibrosis, and lung cancer, emerging respiratory diseases, particularly those caused by members of the coronavirus family, have contributed to a significant number of deaths on a global scale over the last two decades. Therefore, several studies have been conducted to optimize the efficacy of treatments against these diseases, focusing on pulmonary drug delivery using nanomedicine. Thus, the development of nanocarriers has emerged as a promising alternative to overcome the limitations of conventional therapy, by increasing drug bioavailability at the target site and reducing unwanted side effects. In this context, nanoparticles composed of chitosan (CS) show advantages over other nanocarriers because chitosan possesses intrinsic biological properties, such as anti-inflammatory, antimicrobial, and mucoadhesive capacity. Moreover, CS nanoparticles have the potential to enhance drug stability, prolong the duration of action, improve drug targeting, control drug release, optimize dissolution of poorly soluble drugs, and increase cell membrane permeability of hydrophobic drugs. These properties could optimize the performance of the drug after its pulmonary administration. Therefore, this review aims to discuss the potential of chitosan nanoparticles for pulmonary drug delivery, highlighting how their biological properties can improve the treatment of pulmonary diseases, including their synergistic action with the encapsulated drug.

## 1. Introduction

Lung diseases are among the top 10 causes of death worldwide. Chronic obstructive pulmonary disease (COPD), lower respiratory tract infections, and lung cancer are the third, fourth, and sixth leading causes of death, respectively [1]. In addition, the COVID-19 pandemic had a significant impact on the increase in deaths associated with respiratory diseases. Since the emergence of SARS-CoV-2, the number of people infected with the virus who have developed severe respiratory symptoms, such as pneumonia and severe acute respiratory syndrome (SARS), has increased exponentially [1,2]. Also, the social distancing and lockdown measures required to control the spread of COVID-19 may have led to a delay in the diagnosis and treatment of non-coronavirus-related lung diseases, increasing the risk of complications and mortality associated with these conditions [3].

Therefore, research and development of pulmonary drug delivery systems (PDDS) has intensified in recent years to optimize the treatment of lung diseases. PDDS offers several advantages, including targeted delivery for local treatment, maintenance of high local drug concentrations to enhance therapeutic efficacy while minimizing systemic side effects, controlled drug release, and improved patient compliance [4]. However, drug delivery to the lungs presents major challenges. The primary challenge is to overcome the mechanical, chemical and immunological defenses of the respiratory tract that prevent inhaled drug particles from entering the lungs, and then to remove or neutralize them after deposition [5,6]. Several strategies have been developed and patented to overcome this problem [7,8,9]. In this sense, nanostructured carriers, due to their reduced size and increased surface-to-volume ratio, can provide effective drug absorption into the lung epithelium and avoid lung clearance. Moreover, nanoparticles can contribute to improve the stability and solubility of the drug, increase its cellular uptake, and reduce potential toxic effects [10].

Among the various types of nanostructures, polymeric nanoparticles have been widely investigated due to their properties of biocompatibility, biodegradability, low toxicity, and scale-up feasibility [11,12,13]. Several natural polymers can be used for the preparation of nanoparticles. However, chitosan (CS) has demonstrated significant potential for drug delivery applications, including pulmonary delivery, due to its intrinsic properties, which include mucoadhesive potential, and antimicrobial, anti-inflammatory, antioxidant, and wound-healing activities [14,15]. Additionally, the FDA has classified this polymer as “Generally Recognized as Safe” (GRAS) and has granted approval for its use in tissue engineering and drug delivery devices [16].

CS nanoparticles have the ability to enhance drug stability, prolong the duration of action, control drug release, optimize dissolution of poorly soluble drugs, and increase cell membrane permeability of hydrophobic drugs. In addition, CS nanoparticles can be functionalized to enhance adhesion to lung cells and direct specific drug delivery to the site of action, thereby minimizing systemic side effects [17]. This advancement in drug delivery technology offers significant potential for treating chronic lung diseases such as asthma and COPD disease, as well as for improving drug delivery for other lung diseases such as bacterial and fungal infections and lung cancer.

In this regard, there are reports that after inhalation, CS nanoparticles could improve drug transport across the mucus layer in a murine asthma model [18]; enhance drug bioavailability and in vivo lung deposition in an animal model aimed at COPD treatment [19]; provide higher inhibitory effect on *P. aeruginosa* biofilm [20] and reduce inflammatory response [21] in the context of cystic fibrosis (CF); improve drug selectivity in lung cancer cells [22]; increase antituberculostatic drug concentration in the lung [15]; and reduce systemic toxicity of drugs used to treat severe COVID-19 infections [23,24,25].

Given the above, this review focuses on the advantages and prospective of CS nanoparticles as pulmonary drug delivery systems, including small molecule drugs, proteins/peptides, and genes for the treatment of local lung diseases. Data collection was conducted through Scopus, Web of Science, Science Direct, PubMed, and Espacenet covering the period from 2013 to 2023.

## 2. Pulmonary Route: Challenges and Opportunities

The pulmonary route overcomes several limitations associated with conventional dosage forms, and offers the opportunity to reduce the administered dose compared to the oral and parenteral routes. This is due to its characteristics, which include a large surface area (100 m^2^), abundant blood supply, high permeability of the thin peripheral epithelial layer (0.2–0.7 µm), low enzymatic activity, and the ability to avoid first-pass metabolism [26]. Regardless of these factors, the large surface area and perfusion of the lung and its thin diffusion pathway from the airspace to the bloodstream allow for local or systemic administration of drugs via the pulmonary route [27]. Therefore, pulmonary drug delivery has become the first choice for the treatment of local diseases such as asthma, cystic fibrosis, and COPD. In addition, the pulmonary route has been used to treat systemic diseases such as diabetes [28].

Despite the great potential for drug release, the pulmonary route faces obstacles that can compromise its therapeutic efficacy for both local and systemic treatments. One major challenge is the clearance mechanisms that drugs encounter when administered via inhalation, which act as primary barriers to drug absorption after pulmonary administration [29]. Among these, mucociliary clearance plays a critical role as the lung’s defense mechanism responsible for eliminating inhaled particles and exogenous substances [30,31].

After inhalation, molecules become entrapped in the mucus layer, which negatively affects their solubility, diffusion across epithelial layers, and their ability to bind to cell surfaces and/or receptors. Thus, drugs that are unable to penetrate the mucus layer are subsequently removed through mucociliary clearance [32]. Therefore, the most important step for optimal drug delivery is to cross the mucus layer to reach the alveolar epithelial layer [33,34]. In this sense, particle properties such as size and surface area are essential elements that influence the overall efficiency of the drug delivery system [35]. Based on this, several studies have investigated the use of CS nanoparticles as a pulmonary delivery system, since this polymer has mucoadhesive properties and can assist drug penetration through the mucus layer by interacting with mucin [14,36,37,38].

In addition, pulmonary surfactant, a lipoprotein complex with amphiphilic properties produced by alveolar cells, plays a role in enhancing the adhesion and agglutination of certain drugs, leading to an increase in their clearance from the lungs [30]. However, recent studies have shown that pulmonary surfactant is not an insurmountable barrier, and can serve as an effective vehicle for delivering both hydrophobic and hydrophilic compounds deep into the lungs [39,40].

On the other hand, pulmonary macrophages pose a significant challenge to the efficacy of drugs released in the lung, because they tend to internalize particles in the size range of 0.5 to 5.0 µm. The endocytosis mechanism is the primary factor that hinders the absorption of certain inhaled drugs in the lungs, especially macromolecular substances [41,42]. Nevertheless, studies have demonstrated the potential of lung macrophages as a therapeutic target [43], particularly for the treatment of tuberculosis [7,44,45]. With this in mind, Pawde and colleagues [46] developed mannose-functionalized CS nanoparticles containing clofazimine for the treatment of drug-resistant tuberculosis. The mannose-decorated nanoparticles contributed to increased recognition by macrophages, facilitating their uptake and consequently the delivery of the drug to the site of *Mycobacterium tuberculosis* infection.

## 3. Chitosan: Physicochemical and Biological Properties

**Figure 1:**
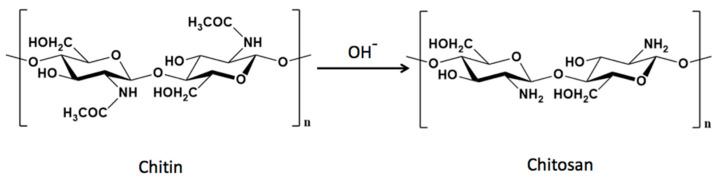
Alkaline reaction for the deacetylation of chitin with the formation of CS. Generally, chitin undergoes a treatment process in which it is exposed to a concentrated NaOH solution at elevated temperatures for an extended period of time, producing chitosan as an insoluble residue.

CS is a linear amino polysaccharide composed of repeating 2-amino-2-deoxy-β-(1,4)-d-glucosamine and 2-acetamide-2-deoxy-β-(1,4)-d-glucosamine units formed by the partial deacetylation of chitin under alkaline conditions (Figure 1). Chitin, in turn, consists of the polymer poly (β-(1,4)-N-acetyl-D-glucosamine), an abundant natural polymer found in the exoskeleton of crustaceans, insects, arthropods, and the cell wall of fungi. Marine chitin, derived from sources such as shrimp, lobster, and crab, is the primary source of commercially available CS [47,48].

Regarding the chemical characteristics, CS is a linear polycationic polymer that has free acetamide groups and hydroxyl functions attached to the glucopyranose rings, which are susceptible to reaction via nucleophilic attack. Thus, selective modification of the free amino groups in CS generates a wide range of functionality for this polymer. Unlike chitin, which has limitations in its use because of its low aqueous solubility and low reactivity, CS is more hydrophilic and has greater potential to be modified due to the aforementioned free amino groups. In addition, while chitin has low biodegradability, CS has excellent degradability and biocompatibility [14,49].

CS is soluble in dilute acidic solutions; however, when the pH exceeds its pKa, CS has a tendency to lose its positive charge and precipitate as a result of deprotonation of the amino groups, rendering it insoluble. This phenomenon is attributed to the fact that most amino groups become neutral at a pH close to 7 [50]. The solubilization process of CS in dilute acid is based on the fact that the amino group combines with hydrogen protons in the aqueous solution, which makes it a positively charged polyelectrolyte. In this way, the cations break the hydrogen bonds originally present between the CS molecules, which causes them to dissolve [48]. In general, the higher the degree of deacetylation of CS, the higher the degree of protonation of amino groups and the easier it is to dissolve. In contrast, the higher the molecular weight of CS, the greater the number of CS–CS hydrophobic contacts which makes dissolution more difficult [51].

It is important to emphasize that the choice of acid type for CS solubilization is a critical parameter that should be tailored to the specific requirements of the intended application. Different acids can cause variations in important physicochemical parameters such as solubility, viscosity, ionic strength and stability [52]. With respect to solubility, different dilute acids have different abilities to protonate the amino groups of CS, which affects its solubility. In this sense, acetic acid is commonly used and provides adequate solubility for CS. CS can also be solubilized in citric acid, although its solubility may be lower compared to acetic acid. On the other hand, formic acid can dissolve CS to some extent, although its solubility may be lower compared to acetic acid. In this case, the solubility may be influenced by factors such as temperature and DDA of CS [53].

The choice of acid can also affect the viscosity of the CS solution. Some acids may result in more viscous solutions. For example, acetic acid tends to increase the viscosity of the CS solution more than lactic acid [54] and less than citric acid [55]. In addition, the type of acid may also influence the ionic strength of the CS solution, which may affect interactions with other compounds or materials. In this regard, it is reported that the ionic strength of the CS solution solubilized in acetic acid tends to be higher than that of the CS solution solubilized in citric acid [56]. The stability of the CS solution, including its shelf life and resistance to degradation, may also be affected by the type of acid used. In this sense, Sikorski et al. (2021) reported that malic acid showed a stronger degradation effect on CS than acetic, lactic or formic acid [57]. Finally, it is interesting to note that the acids used to solubilize CS can interact electrostatically with positively charged amino groups on CS, which may allow the formation of nanoparticles. In this regard, there are reports on the formation of CS nanoparticles using, for example, aspartic acid [52] and citric acid [58] as crosslinking agents.

The crystallinity of CS consists of the ratio between the crystalline and amorphous fractions of this biopolymer, which is quantified as the crystallinity index (CI), determined by the ratio between the characteristic peaks in X-ray diffraction (XRD) [59]. CS, in its solid state, is a semi-crystalline and polymorphic biopolymer, which is found in three polymorphic phases with different degrees of crystallinity, depending on the source and the arrangement of the chains. In this aspect, it is worth pointing out that the quantitative evaluation of CI is extremely important since this parameter affects the swelling, porosity, hydration, and absorption properties of CS [60].

The molecular weight of CS, in turn, is related to the number of monomeric units of the biopolymer, and its evaluation is important because this property is directly related to properties such as viscosity and solubility. Based on molecular weight, CS can be classified as low, medium, or high molecular weight. The majority of commercially available CS typically have a molecular weight in the range of 50 to 2000 kDa [49,61]. Studies have already demonstrated the effect of MW on the uptake of CS nanoparticles in different cell lines. Using the A549 cell line (lung carcinoma epithelial cells), a higher uptake was observed for CS with a high MW [62,63].

Regarding the percentage of N-deacetylation of CS, this property can be defined as the degree of deacetylation (DDA). Commercial CS generally has a DDA in the range of 70 to 95% [64]. It is worth noting that the DDA is an essential factor in determining the physicochemical properties of CS because it represents the amount of NH_2_ groups that can be protonated in an acidic medium and is therefore closely related to its solubility [65]. In addition, DDA determines several physical, chemical, and biological properties of CS, such as viscosity, mechanical behavior, biodegradation, mucoadhesive property, and antimicrobial activity [48,50,66]. In this regard, there are reports that the mucoadhesive and antibacterial properties of CS increase with increasing DDA [67,68].

In addition to greater solubility in acidic environments, degradability, biocompatibility, and low toxicity, CS presents many biological activities, such as antimicrobial, antioxidant, anti-inflammatory, and anticancer. These properties make CS a polymer with unique potential, and have contributed to its investigation as a drug delivery device.

Concerning antimicrobial activity, there are several hypotheses respecting the mechanism of action of CS. One of them is that the interaction between the positively charged CS molecules and the negatively charged bacterial cell membrane causes the rupture of bacterial biofilms and the leakage of cellular components [17,61,69]. It is also hypothesized that CS acts as a chelating agent, which inhibits the production of toxins and the growth of microorganisms. Also, CS can bind with bacterial DNA to enter the nucleus of microorganisms, which inhibits mRNA synthesis and consequently protein synthesis [70]. The antimicrobial activity of chitosan is influenced by several intrinsic factors, including its origin (derived from crustaceans, insect shells, or fungi), concentration, MW, chain configuration, and DDA, as well as extrinsic factors such as environmental pH, and the specific type of microorganism and its susceptibility [50,71]. Concerning pulmonary pathologies, there are reports that CS-based nanoparticles are able to enhance antimicrobial activity against *M. tuberculosis* [72] and *Pseudomonas aeruginosa* [20], which is associated with lung infection in patients with cystic fibrosis.

Regarding antioxidant activity, CS has already demonstrated the ability to remove excess free radicals in the body, increase the activity of antioxidant enzymes and inhibit lipid peroxidation activity. In this context, the molecular weight and the DDA of CS are also directly related to the higher or lower activity of the polymer [73]. It has been shown that the antioxidant properties of CS are greater when the molecular weight is lower and/or the DDA is higher [74,75,76]. In this way, there are reports that CS with a molecular weight of approximately 5.0 kDa exhibits stronger antioxidant properties at lower effective concentrations against the DPPH radical [77]. Since excess free radicals may be involved in pathological processes such as different types of cancer, including lung cancer, Kumar et al. [78] evaluated the antioxidant and antitumor potential of CS nanoparticles containing the flavonoid naringenin (CSNPs/NAR) against A549 cells. As a result, the authors observed that the free radical scavenging activity of CSNPs/NAR was significantly higher than that of the free drug.

The immunostimulatory effect of CS has been attributed to the presence of N-acetyl-D-glucosamine groups, which act by stimulating inflammatory cells such as PMN neutrophils, macrophages, and fibroblasts [79,80]. In addition, CS has been reported to promote the production of transforming growth factor β (TGF-β) and platelet-derived growth factor by human macrophage-derived monocytes involved in the inflammatory response [81]. Specifically, high molecular weight CS increases the production of interleukin-1 (IL-1), tumor necrosis factor-alpha (TNF-α), granulocyte-macrophage colony-stimulating factor (GM-CSF), nitric oxide (NO), and interleukin-6 (IL-6) in macrophages, contributing to the anti-inflammatory effect. For low molecular weight CS, Zheng and colleagues [82] have reported its ability to exhibit immunostimulatory activity via the activation of NF-κB and AP-1 pathways in the macrophage cell line (RAW 264.7).

On the other hand, CS oligomers stimulate the release of TNF-α and IL-1β, highlighting their immunostimulatory effect [83]. Similarly, the study by Chung and colleagues [84] demonstrated the ability of low molecular weight CS oligosaccharides (MW < 1 kDa) in reducing the generation and release of Th2 cytokines (IL-4, IL-5, and IL-13), along with the proinflammatory cytokine TNF-α, in both IgE antigen-stimulated rat basophilic leukemia RBL-2H3 cells and in mice with an allergic asthma model sensitized and challenged with OVA.

The anticancer activity of CS depends on the molecular weight, degree of acetylation, tumor type, source, and CS derivatives [85]. The antitumor mechanism is related to the inhibition of proliferation and induction of apoptosis of tumor cells. At the same time, CS activates the immune system, increasing the ability of immune cells to recognize and reduce tumor cells [86]. Literature data emphasize that the anticancer effect of CS on cancer cell lines tends to decrease as its molecular weight increases, which is due to the diffusion limitation caused by the high viscosity of high molecular weight CS samples [87]. It is important to emphasize that the presence of free protonable amino groups in the CS chain favors the flexibility of its structure. This property allows for the easy development of modified and functionalized CS, transforming it into a versatile polymer capable of producing nanoparticles highly specific for tumor targeting [88].

## 4. Preparation of Chitosan-Based Nanoparticles

Several methods have been developed for the preparation of CS nanoparticles, and the basic approaches used consist of emulsification, precipitation, ionic or covalent crosslinking, or combinations of these techniques [89]. The first method described for the preparation of CS nanoparticles was based on covalent crosslinking by reacting the amino group of CS and the aldehyde group of a crosslinking agent, such as glutaraldehyde [90]. In this process, an emulsion is produced by mixing an aqueous solution of CS with an oil phase composed of Span 80 (stabilizer), toluene and glutaraldehyde. Although this method produces nanoparticles of small size and narrow distribution, it is no longer used because glutaraldehyde has been found to cause apparent toxicity and problems with drug integrity [91].

CS nanoparticles can also be prepared using precipitation-based methods, including phase inversion precipitation and desolvation. Phase inversion precipitation begins with an emulsification process that requires the use of an aqueous CS solution with a stabilizer (poloxamer) and an organic phase (dichloromethane and acetone). A high-pressure homogenizer is then used to obtain nanometric emulsion droplets. Finally, the nanoparticles are precipitated by evaporating the dichloromethane at low pressure and room temperature [92,93]. Desolvation, also known as phase separation or simple coacervation, consists of the coalescence of two water-in-oil emulsions, which promotes the precipitation of nanoparticles. In this method, a mixture of liquid paraffin and sesquioleate is used as the continuous phase for the two emulsions, one with CS and the other with NaOH. After combining the two emulsions, the NaOH diffuses into droplets that reduce the solubility of the CS, leading to the formation and precipitation of nanoparticles [91,94]. These precipitation methods are not widely used due to the need for organic solvents and high-energy homogenization processes. In addition, CS nanoparticles produced using precipitation methods are typically larger than 600–800 nm [95].

**Figure 2:**
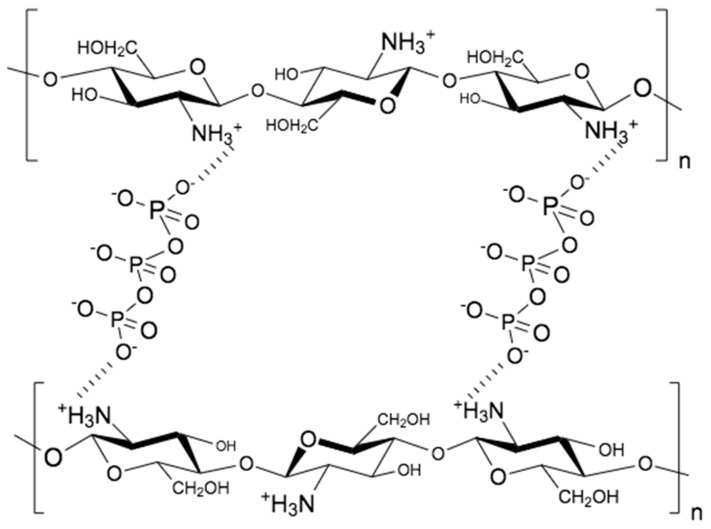
Schematic representation of the chitosan/TPP interaction in the ionic gelation process. The interaction occurs between the protonated amino groups of chitosan and the negative charges of TPP after its ionization in an aqueous medium.

The ionic gelation technique has been the most widely used since its introduction by Calvo et al. [96] due to its simplicity, low cost, environmental friendliness, and potential scalability [97]. Ionic gelation involves electrostatic interactions between positively charged primary amino groups of CS and negatively charged polyanions that act as crosslinking agents [98]. An aqueous solution of the cross-linking agent, e.g., sodium tripolyphosphate (TPP), is added to the acidic aqueous solution (pH 4–6) of CS via dripping. The nanospheres are formed spontaneously after the two solutions are mixed by the inter- and intramolecular bonds between the phosphate groups of the TPP and the protonated amino groups of the CS [96,99,100] (Figure 2). In addition to the aforementioned advantages, this method allows the final size of the nanoparticles to be adjusted by changing the CS/TPP ratio, which directly affects the efficiency and delivery of the drug encapsulation [101]. Although ionic gelation has many advantages, a limitation of the technique is that the particles are formed by electrostatic interactions, which can cause destabilization of the system when pH changes occur. In addition, this method typically produces nanoparticles of large size (100–400 nm) and with a high degree of polydispersity [102,103].

The reverse micellar method (microemulsion), like emulsification and crosslinking, is based on covalent crosslinking and uses reverse micelles as nanoreactors. In this technique, crosslinkers (molecules with at least two reactive functional groups) allow for the formation of bridges between CS chains, producing aggregates of interconnected CS polymers. Normally, an aqueous phase containing CS and glutaraldehyde is mixed with an organic phase composed of an organic solvent and a lipophilic surfactant, such as sodium bis-(2-ethylhexyl) sulphosuccinate [104]. In the production of CS nanoparticles, a limiting factor of most methods is the difficulty of controlling the size distribution of the particles, a limitation that the reverse micellar method has overcome since nanoparticles with a narrow size distribution are produced [105]. Furthermore, compared to the ionic gelation method, for example, the microemulsion technique produces smaller nanoparticles (equal to or smaller than 100 nm). However, although glutaraldehyde is one of the most effective cross-linking agents for obtaining CS nanoparticles, its cytotoxicity is limits its biomedical applications. This problem raises the need to study effective and safe crosslinking agents for application in the reverse micellar method, such as genipin [106,107,108].

Currently, the study of environmentally friendly preparation techniques is being increasingly explored. In this context, the spray drying method for the preparation of CS nanoparticles stands out. In this technique, CS is usually dissolved in aqueous acetic acid, and the nanoparticles are formed by passing this solution through a nozzle with air temperatures between 120 °C and 150 °C. The properties of CS nanoparticles obtained via the spray drying technique depend on various operating parameters, including nozzle size, flow rate, and inlet and outlet temperatures [109]. Disadvantages of this method include longer processing time, large size of the particles produced, and the fact that it is not suitable for thermosensitive drugs [110].

**Figure 3:**
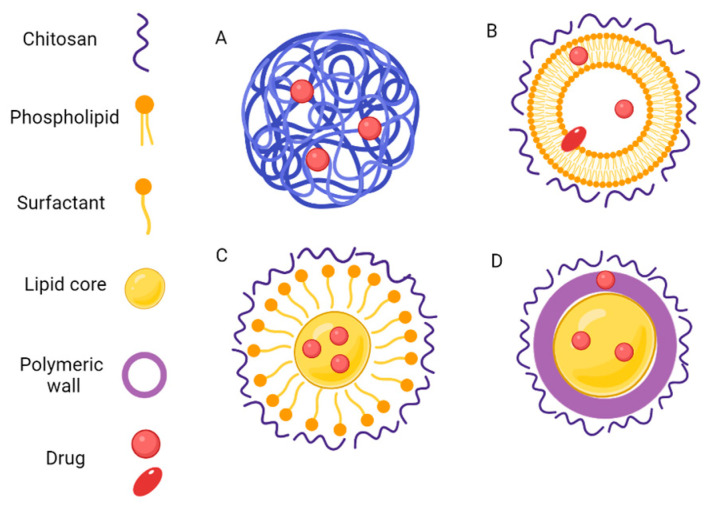
Examples of some CS-based nanoparticles, which can be essentially formed by CS or prepared from CS-coated phospholipidic, lipidic or polymeric nanoparticles. (**A**): CS nanosphere, with drug dispersed in polymer matrix; (**B**): CS-coated liposome. In this case, hydrophilic drugs can be encapsulated in the aqueous compartment, hydrophobic drugs in lipid bilayers and amphiphilic drugs between these two compartments.; (**C**): CS-coated solid lipid nanoparticle with the drug encapsulated in the oil core; and (**D**): CS-coated polymeric nanocapsule, where the drug can be encapsulated within the oil core or adsorbed to the polymeric wall.

In addition, CS can be used to coat the surface of nanostructures such as liposomes [111], solid lipid nanoparticles [112], and polymeric nanoparticles [50] (Figure 3). For this purpose, CS can be introduced during the preparation of the nanoparticles or at a later stage, after the formation of the nanostructures. In this case, the CS solution can be dripped onto the nanoparticle suspension under moderate agitation [113]. In the case of polymeric nanoparticles, the coating could be achieved by the entanglement of the polymer chains, resulting in a coated core structure. On the other hand, the coating can also be achieved by the interaction between the negative charges present on the lipid or polymeric nanoparticle surface and the positive charges present on the CS chain [114,115].

## 5. Chitosan-Based Nanoparticles for Pulmonary Delivery

The application of CS nanoparticles for pulmonary delivery has faced challenges, mainly due to their tendency to aggregate and be exhaled. To address these issues and to create a stable and solid formulation, the use of particle engineering techniques like freeze-drying [116] and spray drying [117] has emerged as a promising tool. In this sense, the use of carriers such as lactose and mannitol are essential to produce nanoparticles as dry inhalable powders with a favorable mean aerodynamic diameter for optimized deposition in the alveoli and to prevent nanoparticle aggregation [118].

**Figure 4:**
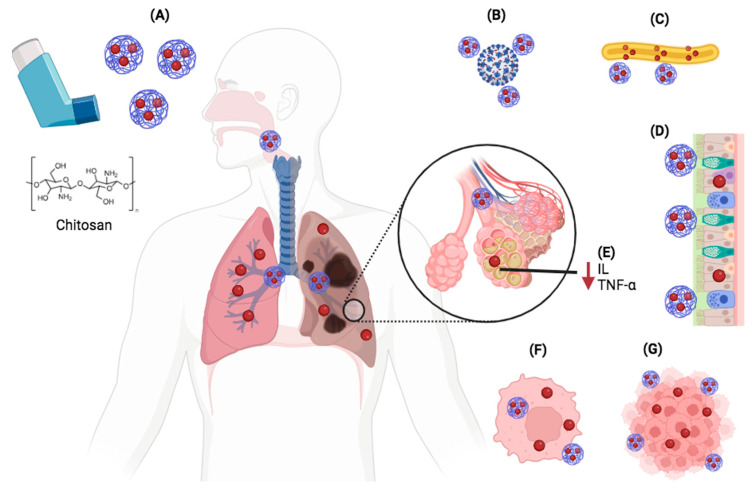
Some important advantages of CS nanoparticles (**A**) in pulmonary drug delivery. Inhaled CS nanoparticles are able to improve antiviral (**B**) and antibacterial (**C**) activities, facilitate drug penetration through the mucus layer (**D**), contribute to anti-inflammatory activity (**E**), and increase the interaction/internalization in specific cells such as macrophages (**F**) and tumor cells (**G**).

Due to the intrinsic properties of CS, such as mucoadhesive properties, and anti-inflammatory and antimicrobial activities, CS nanoparticles (Figure 4A) are able to provide numerous advantages for local drug delivery in the lung. In this sense, nanoparticles can enhance the antiviral (e.g., anti-SARS-CoV-2 activity) (Figure 4B) and antibacterial (e.g., against *M. tuberculosis*) (Figure 4C) activities of encapsulated drugs. In addition, they facilitate the penetration of the drug through the mucosal layer (Figure 4D), can enhance the anti-inflammatory activity of the drug (Figure 4E), and allow for greater interaction/internalization in specific cells, such as macrophages (Figure 4F) and tumor cells (Figure 4G).

Due to its unique properties and numerous advantages, several studies have been conducted aiming at pulmonary delivery of drugs encapsulated in CS-based nanoparticles. Summarized data are presented in Table 1.

### 5.1. Pulmonary Chronic Diseases

Chronic respiratory diseases basically are correlated to asthma, chronic obstructive pulmonary disease (COPD), idiopathic pulmonary fibrosis (IPF), cystic fibrosis (CF), and lung cancer [134]. In this sense, several studies have been conducted, demonstrating that CS nanoparticles are able to increase the efficacy and safety of encapsulated drugs aimed at the treatment of these pathologies.

#### 5.1.1. Asthma

Asthma is a complex disease involving irreversible airway obstruction, airway hyperresponsiveness, and chronic airway inflammation that remodels the airway wall [135]. Treatment focuses on symptoms and includes pharmacologic bronchodilators, beta-2 agonists, and anti-inflammatory glucocorticosteroids. Although asthma attacks are well controlled with glucocorticoids and long-acting β-agonists, high-dose administration of these drugs has been shown to be clinically ineffective and potentially harmful, while poorly managed inflammation can cause sudden death in severe asthma. Therefore, alternative anti-inflammatory interventions to conventional treatments need to be developed with the goal of controlling airway remodeling and hypersensitivity without the risk of serious adverse effects [18].

**Figure 5:**
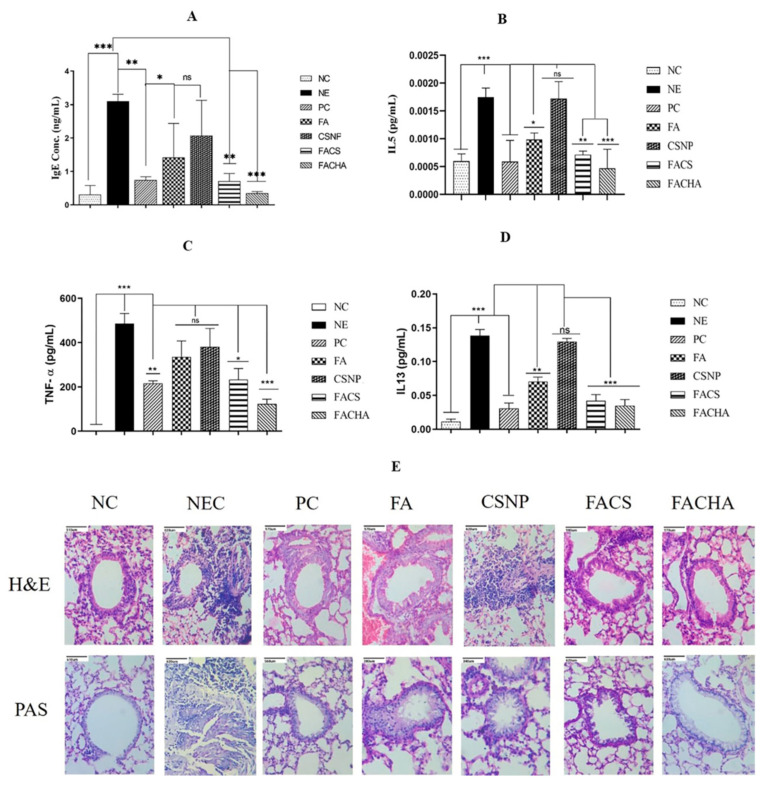
Effect of FACHA on serum levels of IgE (**A**), IL5 (**B**), TNF-α (**C**) and IL13 (**D**) quantified using ELISA after pulmonary administration in an OVA-induced mouse model of asthma. These cytokines play distinct but related roles in the pathogenesis of asthma, contributing to the inflammation, bronchial hyperreactivity, and excessive mucus production that characterize the disease. (**A**) shows that, compared to FACHA treatment, FA demonstrated a more limited ability to reduce serum IgE levels. Also, FACHA treatment showed remarkable efficacy (*p* ≤ 0.001) in reducing IL5 (**B**), TNFα (**C**), and IL13 ((**D**) levels. This provides compelling evidence for the ability of the formulation to alleviate the asthmatic condition in OVA-sensitized mice. (**E**) shows histopathologic sections of lungs from treated mice. As can be seen, the FACHA-treated group showed normal morphological features, suggesting its protective effect against excessive mucus secretion and inflammatory cell infiltration in lung tissue. These findings support the enhanced efficacy of FACHA compared to pure FA, which is attributed to the mucoadhesive nanocarrier properties that enhance drug retention and facilitate transport across the pulmonary barrier. NC: normal control (0.9 N saline); NE: negative control (OVA sensitization); PC: positive control (OVA sensitization followed by budesonide treatment (9.50 mg/m^3^); FA: ferulic acid; CSNP: unloaded CS nanoparticles; FACS: ferulic acid-loaded CS nanoparticle; FACHA: hyaluronic acid functionalized ferulic acid-loaded CS nanoparticle.; H&E: hematoxylin and eosin; PAS: periodic acid Schiff stain; Statistical significance—*** (*p* ≤ 0.001), ** (*p* ≤ 0.01), * (*p* ≤ 0.05), ns (*p* greater than 0.05) [18]. Reproduced with permission from Dhayanandamoorthy et al., *Int. J. Pharm.*; published by Elsevier, 2020.

Dhayanandamoorthy et al. [18] developed CS nanoparticles functionalized with hyaluronic acid (HA) and loaded with ferulic acid (FA), a potent anti-inflammatory drug. The nanoparticles (FACHA) were aerosolized using the Aeroneb^®^ vibrating mesh nebulizer. In vivo toxicity studies confirmed the safety of FACHA nanoparticles. At the same time, when used for asthma prophylaxis, FACHA nanoparticles were able to attenuate inflammation, hypersensitivity and airway remodeling in mouse models of ovalbumin (OVA)-induced asthma (Figure 5). Compared to free FA, the nanostructured systems exhibited superior in vivo therapeutic indices due to the encapsulation of the drug by the HA-functionalized CS nanoparticles, which, in combination with the administration with the vibrating mesh nebulizer, were able to promote a better deposition and an improved therapeutic index of HA.

Budesonide (BUD) is poorly bioavailable in the lung and is used in the treatment of asthma. Ahmad et al. [19] developed a BUD-loaded CS-PLGA nanoparticle to treat asthma and improve the solubility of budesonide. The authors demonstrated BUD-NP lung deposition and budesonide penetration. In addition, Cmax and AUC were higher with inhalation compared to both oral and i.v. treatment groups. Furthermore, they demonstrated that the improvements in BUD absorption resulted from the induction of intercellular tight junction openings within the lung epithelium, an effect facilitated by CS.

Further, CS NPs were used for pulmonary delivery of baicalein, a flavonoid isolated from the roots of *Scutellaria baicalensis* with cytoprotective, anti-inflammatory and myorelaxant properties. The baicalein-CS-NP’ size (285 ± 25 nm) were in the ideal range for pulmonary delivery (50–500 nm). In this regard, the literature indicates that particles smaller than 50 nm may be expelled with exhalation, and that particles larger than 500 nm may be deposited in the upper respiratory tract. After nebulization, baicalein-CS NP controls eosinophilic inflammation by downregulating IL-5 levels. Also, NP-controlled airway hyperresponsiveness and early phase of immune-allergic and allowed for a better-managed inflammation and the late phase of immune-inflammatory response in airways [119]. Several studies have already demonstrated the immunomodulatory and anti-inflammatory activities of CS [79,80,81,82,83,84]. Therefore, the biological properties of this polymer may also be responsible for the activities observed in this study.

#### 5.1.2. Chronic Obstructive Pulmonary Disease (COPD)

COPD is a progressive inflammatory disease of the lung parenchyma and small airways. The progression leads to luminal obstruction, airway wall thickening due to increased mesenchymal cell proliferation and matrix molecule deposition, and airway fibrosis. Taken together, they results in decreased lung function [112]. Anti-inflammatory therapies are used to reduce oxidative stress [112] with the goal of preventing lung damage, alleviating symptoms, treating complications, and promoting patient health [121]. Corticosteroids, including BUD, are currently used [91].

BUD, a BCS Class II drug was loaded into CS NP via ionic gelation using poly (vinyl alcohol) (PVA) as a surfactant. The NPs were spherical with average size between 363 and 543 nm and had zeta potential higher than +36 mV, indicating a positive effect on colloidal stability and mucoadhesiveness. Due to BUD amorphization in the nanoparticles, the authors reported an improved drug release in in vitro release studies. [121].

CS can be used in the production of supramolecular structures built with other macromolecules. Black phosphorus quantum dots (BPQDs) were associated with PEGylated CS nanospheres to deliver amikacin (AM). This drug is the first-line treatment for pulmonary infections in COPD patients. However, the lung uptake of AM is limited when administered intravenously, highlighting the need to improve the efficacy of drug therapy while reducing potential adverse effects. The nanostructure exhibited mucoadhesive properties that facilitated penetration through the mucus layer. In addition, the rapid degradation of BPQDS resulted in higher drug release due to the dissociation of PEGylated NP. Therefore, AM was able to rapidly disrupt the viability of *P. aeruginosa* biofilm. The results also showed an alleviation of airflow obstruction in a COPD mice model [122].

#### 5.1.3. Pulmonary Fibrosis

Pulmonary fibrosis is a disease characterized by the remodeling and destruction of lung tissue. Management of pulmonary fibrosis is the goal of treatment, but there is no cure. N-acetylcysteine, corticosteroids and cytotoxic agents are the current drugs administered. In this regard, in an interesting study, the authors demonstrated that the intratracheally inhalable nifedipine, a calcium channel blocker, loaded in CS-PLGA NP could be a promising tool for the treatment of pulmonary fibrosis. The nanoparticles exhibited a spherical shape at the nanoscale (226.46 nm), an entrapment efficiency of 61.8%, and prolonged drug release over 24 h. In addition, these nanoparticles exhibited an enhanced capacity for in vitro lung deposition as evidenced by their mass median aerodynamic diameter (1.12 µm). Pharmacokinetic studies showed significant improvements in bioavailability, with a remarkable 3.68-fold increase compared to oral suspensions and a 2.36-fold increase compared to intratracheal suspensions. In addition, the engineered nanoparticles showed a remarkable reduction in lung fibrotic and oxidative stress markers, similar to the normal control group. Notably, they also demonstrated the ability to correct abnormalities in the TGF-β/β-catenin pathway [123].

#### 5.1.4. Idiopathic Pulmonary Fibrosis (IPF)

IPF is a type of pulmonary fibrosis, which is characterized by persistent fibrotic lung fibroblasts and the excessive production of type I collagen-rich matrix. This disease is associated with progressive deterioration of respiratory function and high mortality since treatment options are still very limited. There is a report that CS NPs may be used to treat IPF as they have shown great potential as a drug delivery system for targeting fibrotic lung fibroblasts [136].

There is a consensus that excessive myofibroblast differentiation significantly contributes to the progression of IPF. This is attributed to the fact that myofibroblasts, characterized by α-smooth muscle actin (α-SMA) expression, generate large amounts of pericellular matrix and fibrogenic cytokines within fibrotic areas, thereby influencing changes in the extracellular mechanical microenvironment of the lung. With this idea in mind, Zhang and co-authors (2019) developed phosphorylcholine-CS nanoparticles (PPCs-NPs) to encapsulate the mutant soluble ectodomain of fibroblast growth factor receptor-2 IIIc (msFGFR2c) protein, which could inhibit the expression of α-smooth muscle actin (α-SMA). The authors observed that α-SMA was significantly reduced when the fibroblasts were treated with PPCs-NPs. Moreover, when tested in vivo, the nanoparticles increased the bioavailability of msFGRF2c after intratracheal administration, improving the protein’s therapeutic efficacy and rat survival rate, which was not observed with the non-encapsulated protein [124].

#### 5.1.5. Cystic Fibrosis

Cystic fibrosis (CF) is caused by impaired mucociliary airway clearance due to a mutation in the cystic fibrosis transmembrane conductance regulator (CFTR) gene. As a result, mucociliary clearance is impaired, leading to increased mucus retention, susceptibility to bacterial infection, inflammatory response, and airway obstruction. There are many treatments for CF, including antibiotics, bronchodilators, and steroids [20,137].

**Figure 6:**
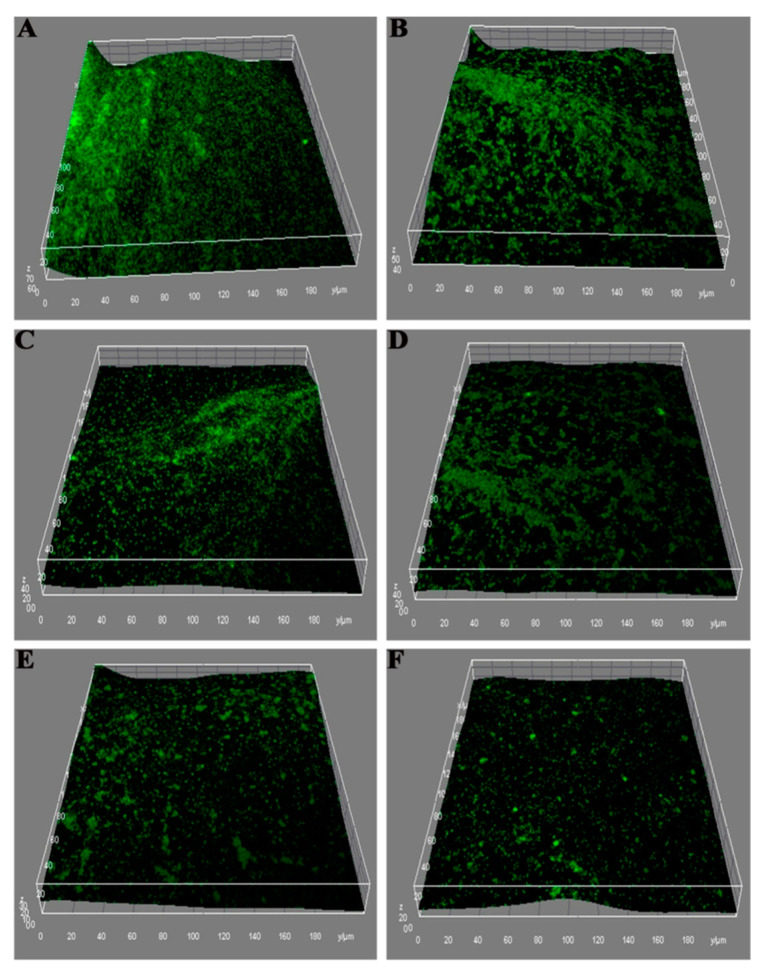
Confocal laser scanning microscopic images of P. aeruginosa biofilm after different treatments: untreated (**A**), CIPR (**B**), CIPR + AgLase (**C**), CIPR-CH-NPs (**D**), CIPR-CH-NPs + AgLase (**E**) and AgLase-CIPR-CH-NPs (**F**). AgLase-CIPR-CH-NPs clearly showed the most potent antibiofilm activity. CIPR: ciprofloxacin; CH: chitosan; AgLase: alginate lyase; NPs: nanoparticles; CIPR-CH-NPs: ciprofloxacin-loaded chitosan nanoparticles; AgLase-CIPR-CH-NPs: alginate lyase functionalized chitosan nanoparticles of ciprofloxacin [20]. Reproduced with permission from Patel et al., *Int. J. Pharm.*; published by Elsevier, 2019.

One of the main focuses of treatment is to combat *P. aeruginosa* infection. Thus, Patel and colleagues [20] investigated the effect of ciprofloxacin-loaded alginate lyase functionalized-CS NP against mucoid *P. aeruginosa* biofilm. The alginate lyase was used to disrupt the bacterial mucus, which enhanced the delivery of ciprofloxacin. The nanoparticles had an average particle size of 205.5 ± 9.0 nm, a positive zeta potential (12.2 ± 2.1 mV), and an encapsulation efficiency of 51%. The particle size is ideal for the rapid diffusion of NP through the thick mucus pores, which effectively delivers the drug to the microbial colonies. Moreover, the powder properties were in accordance with the requirements for PDDS due to the fair particle flow with an angle of repose of 33.4°, an aerodynamic MMAD of 2.69 ± 0.03 µm, and an FPF of 38 ± 2.7%, which are satisfactory to achieve sufficient lung deposition of NPs and their stability. The NPs showed great antimicrobial and anti-biofilm inhibition potential (Figure 6), supporting that nanoparticles are able to disrupt the biofilm and increase bacteria sensitivity to antibiotic [20].

As described, CF arises from genetic mutations within the CFTR gene, which encodes a chloride and a bicarbonate channel dependent on cAMP. This channel is primarily found in the apical membrane of secretory epithelial cells. The impaired chloride secretion mediated by CFTR is associated with increased sodium absorption through the epithelial sodium channel (ENaC).

With this concept, Kolonko et al. [125] developed a surface with wtCFTR-mRNA- CS nanocapsules-loaded capsaicin to normalize the CFTR function. The authors hypothesized that the association between transcript therapy and capsaicin may benefit CF treatment in two complementary ways: first, wtCFTR mRNA may increase chloride secretion in epithelial cells, and second, capsaicin may increase transfection efficiency while causing a secondary reduction in ENaC activity. The nanotechnology was capable of restoring the CFTR function in the CF cell line (CFBE41o) after transfection with wtCFTR-mRNA. These results showed the potential of delivering mRNA for lung treatments. Regarding the effect of capsaicin in increasing transfection, the authors contextualize that this alkaloid is capable of reversibly opening the tight junctions of epithelial cells. It is important to note that this property can also be conferred by CS [14,15,16].

### 5.2. Lung Cancer

Currently, although chemotherapy is still the mainstay of treatment for advanced lung cancer, most traditional chemotherapeutic drugs share the same limitations, including lack of targetability, low bioavailability, and severe side effects due to non-selective delivery of cytotoxic agents that affect healthy cells [22,138]. Therefore, CS nanoparticles have being widely studied as potential drug carriers in cancer therapy, mainly because of their properties of mucoadhesiveness, controlled release, drug targeting, increased permeability and uptake into tumor cells, in addition to their immunomodulatory effect [61,86,139,140].

Cationic nanocarriers are of particular importance in the field of drug delivery, as they have demonstrated the ability to specifically target the tumor vasculature, resulting in increased efficacy of anticancer activity. Based on this, Kamel and co-workres [22] developed a CS-doped self-assembled lecithin-based cationic NP (LeciPlex) loaded with resveratrol. The aim of the study was to improve the solubility and anticancer efficacy of resveratrol, as well as to explore the pulmonary delivery of LeciPlex. The authors reported improved anticancer effects, low toxicity, and increased selectivity of resveratrol-loaded LeciPlex against the A_549_ cell line (lung cancer) compared to unloaded drug.

**Figure 7:**
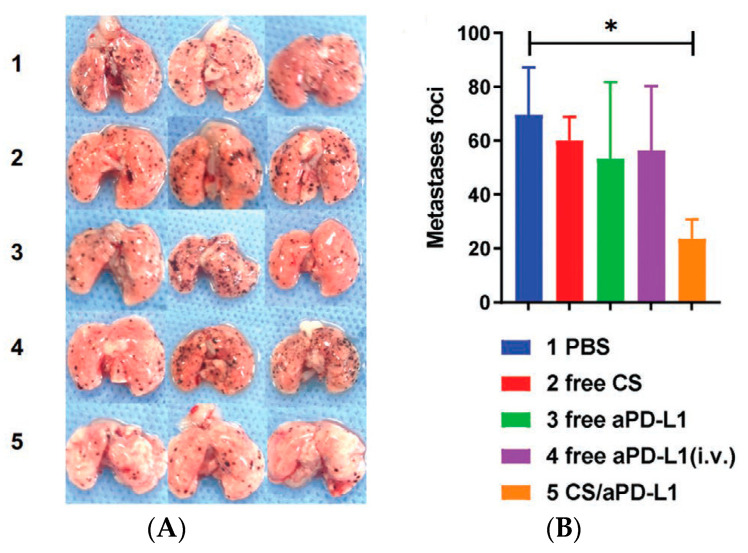
Photos of lungs collected from mice after 15 days of different treatments (**A**). Number of lung metastatic foci on the lung surface after treatments (**B**). In contrast to the other groups, mice treated with CS/aPD-L1 showed a marked decrease in the quantity of lesions. This strongly suggests that the inhalation of CS/aPD-L1 effectively inhibits lung metastasis. PBS: phosphate-buffered saline. CS: chitosan; aPD-L1: anti-programmed cell death protein ligand 1; i.v.: intravenous; * *p* < 0.05 [128]. Reproduced with permission from Jin et al., *Adv. Mater.*; published by John Wiley and Sons, 2021.

In the study developed by Jin et al. [128], inhaled CS NP loaded with the anti-programmed cell death protein ligand 1 (aPD-L1) were prepared to treat lung cancer. The CS was used to enable transmucosal delivery. The authors reported that inhalation of CS nanoparticles with aPD-L1 promoted the rapid accumulation of aPD-L1 in lung metastasis due to the enhanced absorption capacity and transmucosal penetration of CS, which significantly reduced the number of metastases in the lungs (Figure 7). Furthermore, CS was observed to act as an adjuvant of aPD-L1 by being able to induce potent cell-mediated immune responses.

### 5.3. Infectious Diseases

#### 5.3.1. Tuberculosis

Tuberculosis (TB) is a *Mycobacterium tuberculosis* (Mtb) lung infection, and the treatment goal is to eliminate the microorganism. There are available chemotherapy containing first-line medications (isoniazid, pyrazinamide, rifampicin, and ethambutol), second-line injectable medicines (streptomycin, amikacin, kanamycin, viomycin, and capreomycin), and other oral medications [141]. WHO recommends daily oral administration of isoniazid (INH), pyrazinamide (PYZ), rifampicin (RIF), and ethambutol for 2 months, followed by INH and RIF for further 4 months. However, if the first-line medicines do not work, it leads to the administration of the second-line medicines, which are more toxic and expensive [141]. Even the new molecules, bedaquiline and delamanid, are toxic [142]. Therefore, the high daily doses administered over a long period of time, as well as the toxic effects of the drugs used, make adherence to treatment, and consequently, the cure of the disease a major challenge. Moreover, there are many Mtb strains multi-resistant to antimicrobial agents. These drawbacks support the lung drug delivery administration for the local TB treatment focusing on controlled and sustained release [143,144].

CS nanoparticles are emerging as a promising alternative for pulmonary drug delivery for the treatment of tuberculosis. CS is widely recognized for its inherent antimicrobial potential. In this context, the presence of a positive charge on the amine group of the glucosamine monomer under acidic conditions is believed to facilitate interaction with negatively charged microbial cell membranes, resulting in the release of its cellular components [61,69,72]. In addition, CS’s interaction with macrophages holds great promise in the fight against pulmonary tuberculosis. Its positive charge enables efficient uptake by macrophages, the key players in the body’s immune response to tuberculosis [46].

In the study by Shaji et al., D-cycloserine (D-CS) was encapsulated in alginate-CS NP via ionic gelation. The NP had a loading efficiency of 98.10 ± 0.24%, a mean particle size of 344 ± 5 nm, and a zeta potential of −42 ± 11.40 mV. The NP demonstrated controlled release behavior, which improved drug bioavailability and reduced dosing frequency, important for enhancing patient compliance. The system demonstrated a respirable fraction, 52.37 ± 0.7%, suitable for deep lung deposition and was well tolerated in rats [145].

**Figure 8:**
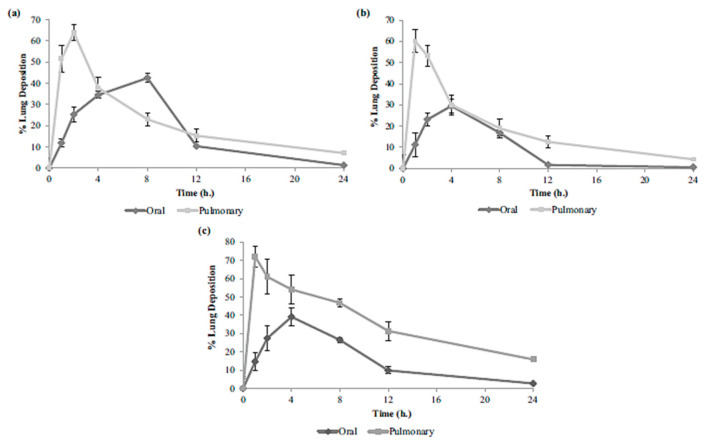
Percentage of the in vivo pulmonary deposition of (**a**) isoniazid, (**b**) pyrazinamide, (**c**) and rifampicin after intratracheal administration of the DPI formulation compared to oral administration of each drug. (values expressed as mean ± standard deviation, *n* = 3). The DPI formulation maintains elevated drug levels in the lungs for a longer period of time compared to standard oral dosing [146]. Reproduced with permission from Chogale et al., *Drug Deliv. Transl. Res.*; published by Springer Nature, 2021.

As described above, treatment success depends on patient compliance, which is strictly dependent on the therapy protocol, including high doses and severe side effects, leading to patient discontinuation. The possible solution could be to deliver the anti-tuberculostatic drugs directly to the lungs. Thus, Chogale and colleagues proposed the development of a Dry Powder Inhaler formulation (DPI) containing three first-line drugs (INH, PYR, and RIF) in a nanostructured form. INH and PYZ were individually loaded into CS NP, and RYF was formulated as a nanocrystal. The nanoparticles were spray-dried, and then formulated with RIF nanocrystals and inhalable lactose. The dry powder inhaler (DPI) showed impressive flow characteristics with a fine particle fraction of 45% and a mass median aerodynamic diameter around 5 µm, indicating favorable lung deposition. In vitro drug release demonstrated sustained release behavior. In vivo studies further demonstrated prolonged pulmonary deposition at higher concentrations compared to oral administration (Figure 8), highlighting the potential advantages of this approach [146]. However, antimicrobial activity was not evaluated.

Prothionamide (PTH) is a second-line drug for TB treatment, with unpredictable absorption, systemic toxicity, and frequent administration, which limit its use. Debnath et al. developed a CS NP loaded with PTH, which was further lyophilized to formulate a DPI. The NPs were spherical with a mean particle size of 314.37 ± 3.68 nm. The DPI formulation presented an aerodynamic particle size of 1.76 µm, which is suitable for PDDS. Furthermore, the drug release followed the Korsmeyer–Peppas kinetic model, and although it had a small size change during storage, the PTH release was not affected. In addition, the CS nanoparticle kept the PTH concentration above the MIC after 12 h, demonstrating the ability of the CS nanoparticle to improve the drug efficacy by increasing the lung tissue concentration [147].

A powder inhaler formulation was developed based on isoniazid and pyrazinamide co-encapsulated in CS nanoparticles via ionic gelation, using TPP as a crosslinking agent.

The aerosol performance of the dry powder was evaluated using the Andersen cascade impactor. The results showed a mass median aerodynamic diameter in the range of 3.3 to 3.5 µm, fine particle fractions in the range of 30% to 44%, and an emitted dose of 92% to 95% for all formulations. Importantly, the respiratory cell lines did not show any toxic responses to the dry powder formulations. In addition, these formulations did not induce alveolar macrophages to produce inflammatory cytokines or nitric oxide, underscoring their safety profile and suggesting their suitability for delivery of tuberculostatic drugs to the respiratory tract [116].

#### 5.3.2. Pneumonia

The pathophysiology of pneumonia involves a complex inflammatory process in the lungs, often triggered by the invasion of pathogens such as bacteria, viruses or fungi. Infection usually begins in the upper respiratory tract and can spread to the alveoli. This leads to the activation of the immune system, resulting in the migration of inflammatory cells such as neutrophils and macrophages to the infected area [148,149]. These cells release cytokines and other inflammatory mediators, which causes an increase in the permeability of the blood vessels in the lungs, leading to extravasation of fluid and cells into the alveoli. The result is an accumulation of inflammatory exudate in the airspaces, which impedes gas exchange and impairs oxygenation. In addition, the presence of pathogens and inflammatory cells in the lungs stimulates coughing and mucus production, contributing to airway obstruction [149].

The standard treatment protocol is to administer drugs orally or parenterally. However, systemic drug delivery can lead to inadequate drug levels in the infected lung area, rapid reduction in plasma levels below therapeutic thresholds, and the emergence of microbial resistance [131]. Inhaled local drug delivery is expected to overcome these barriers. In this regard, the development of PDDS-based chitosan could be beneficial due to its antimicrobial properties [20].

Gallium [Ga(III)] is an iron mimetic metal that has been used in the treatment of several pathologies, including cancer and autoimmune diseases. While Ga(III) bears a remarkable chemical resemblance to Fe(III), it remains unreactive under physiological conditions and thus cannot participate in redox processes. As a result, Ga(III) could act as a “Trojan horse” by replacing Fe(III) in iron-dependent enzymes, thereby suppressing their critical role in bacterial metabolism. In this sense, the potential of this metal against *P. aeruginosa* pneumonia has already been demonstrated in mice. Based on this, Costabile and colleagues [130] developed hyaluronic acid/CS nanoparticles for pulmonary delivery of Ga(III). The developed powder showed adequate in vitro aerosol performance and exhibited sustained release behavior in lung fluids. Satisfactory tolerability in human epithelial bronchial cells (16HBE14o-) and effective antimicrobial properties were also observed. Intratracheal insufflation of the dry powder in rats resulted in a significant improvement in Ga(III) retention in the lung, accompanied by reduced plasma and urine Ga(III) concentrations, compared to gallium nitrate solution.

Gentamicin (GM), a widely used aminoglycoside antibiotic, has found application in the treatment of pneumonia. However, the efficacy of GM is limited by its low bioavailability and potential adverse toxic effects, such as ototoxicity and nephrotoxicity. To optimize the treatment of pneumonia with this antibiotic, GM-loaded in CS/fucoidan NP was developed for pulmonary delivery. The use of fucoidan was based on its ability to scavenge reactive oxygen species generated by GM. The NPs were in the range of 270–300 nm, exhibited a positive zeta potential, and the GM entrapment was higher than 91%. Intratracheal administration of GM-loaded NP improves antimicrobial efficacy and eliminates systemic toxicity [131].

#### 5.3.3. COVID-19

COVID-19 is associated with various symptoms that can range in severity, from flu-like symptoms to pneumonia, acute respiratory syndrome, multiple organ failure and death [26]. Currently, there are no direct-acting antiviral drugs that are fully effective against SARS-CoV-2 for all age groups and non-hospitalized patients. At the same time, most of the drugs used to treat COVID-19 are administered orally, and only a small portion of the drug reaches the lungs due to low bioavailability or gastrointestinal degradation [150].

Because SARS-CoV-2 primarily targets the lungs, the International Association for Aerosols in Medicine (ISAM) has issued a call for research focused on the development of inhalable therapies for this disease [26]. Inhaled treatment of COVID-19 can be extremely beneficial for patients, as pulmonary administration ensures higher drug concentrations in the lungs and blood with lower drug doses compared to the oral route, which means minimal or no side effects with better therapeutic outcomes [4,150].

In addition, the use of polymeric NP for the treatment of COVID-19 is being extensively explored due to their unique physicochemical properties, such as prolonged blood circulation time, reduced side effects, ability to protect therapeutic agents from degradation, and higher stability. In particular, CS nanoparticles have a great potential for application in the treatment of COVID-19, considering the characteristics of this biopolymer, which include low toxicity, biodegradability, suitable physical properties, and mucoadhesive property. In particular, when CS is positively charged and interacts with the mucosa, it is able to open the tight junctions between cells, thereby increasing drug permeation [151,152]. There are also reports that CS has anti-SARS-CoV-2 activity [153]. Thus, the pulmonary administration of CS nanoparticles for the treatment of COVID-19 is an area with great potential for development and should be increasingly explored.

**Figure 9:**
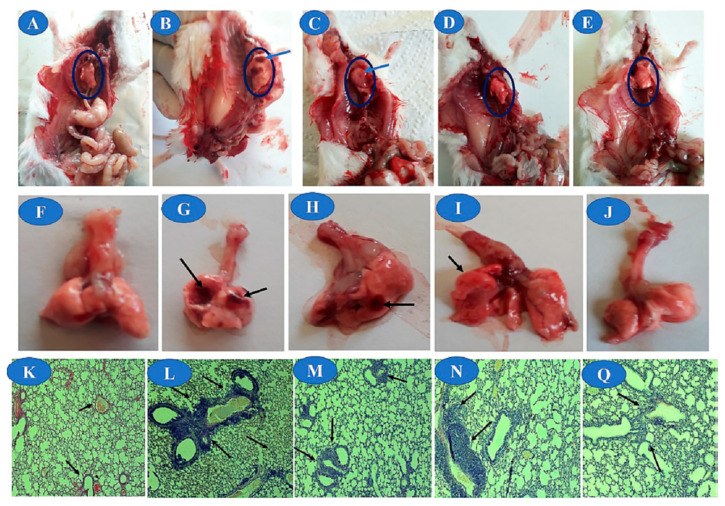
Images of dissected mice, highlighting the alveoli (**A**–**F**). (**A**) Control. (**B**) Oleic acid induced model. (**C**) Treatment with free capsules. (**D**) Treatment with nanoparticles containing extract of chamomile flowers + CUR. (**E**) Treatment with nanoparticles containing SIL. + CUR. Imagens of individual lungs (**F**–**J**). The arrows indicate significant functional changes. (**F**) Control. (**G**) Oleic acid-induced model. (**H**) Treatment with free capsules. (**I**) Treatment with nanoparticles containing extract of chamomile flowers + CUR. (**J**) Treatment with nanoparticles containing SIL. + CUR. Histopathological analysis. (**F**) Control. (**G**) Oleic acid-induced model. (**H**) Treatment with free capsules. (**I**) Treatment with nanoparticles containing extract of chamomile flowers + CUR. (**J**) Treatment with nanoparticles containing SIL. + CUR. (**K**) Control. (**L**) Oleic acid model. (**M**) animal treated by free capsules. (**N**) Animal treated by Encap. Cham. + CUR. (**Q**) Animal treated by Encap. SIL. + CUR. SIL.: silymarin CUR.: curcumin [133]. The co-encapsulation of SIL. + CUR. completely changed the histologic profile and improved the tissue histoarchitecture. Reproduced with permission from Hanafy et al., *Int. J. Biol. Macromol.*; published by Elsevier, 2022.

In the study conducted by Hanafy et al. [133], the polyphenols silymarin (from extract of chamomile flowers) and curcumin were loaded into CS-coated albumin NP and administered via an inhalable delivery system. The NPs are able to reduce the levels of IL-6 and c-reactive protein in vitro, both of which are clinically used as markers for the assessment of severe pulmonary infectious diseases. NPs also showed anti-SARS-CoV-2 activity in vitro. In addition, after in vivo administration to rats, NPs improved their lung histopathology (Figure 9).

In 2020, Bioavanta-Bosti presented the development of CS nanoparticles in aerosol form (Novochisol™, Monthey, Switzerland). These nanoparticles allow for the encapsulation of various drugs for the treatment of severe COVID-19 infections. According to the researchers, the aerosols containing CS nanoparticles enable adhesion and helps in targeting drugs to the epithelial tissues of the lung. In addition, they provide controlled release due to the diffusion and slow degradation of CS, resulting in high local drug concentrations without systemic distribution, thereby reducing toxicity [23,24,25].

Tu and co-workers developed inhaled heparin-loaded CS nanoparticles for the treatment of SARS-CoV-2 infection [154]. In this case, the polyanionic heparin is able to crosslink with the cationic CS through electrostatic interactions to form the stable and spherical-shaped nanoparticles. It was found that the CS nanoparticles with heparin were able to neutralize SARS-CoV-2 and Delta mutant strains and inhibit infection in lung tissue. In addition, production of the nanoparticles is simple and fast, and pulmonary administration reduces unwanted exposure of the drug to other organs.

Finally, it is important to note that CS has poor solubility in neutral and basic media, as well as in organic solvents, which may limit its use. To solve this problem, CS derivatives have been synthesized. These derivatives are synthesized with reactions occurring in the amino group of C2 or in the hydroxyls of C3 and C6, such as N-reducing alkylations, N-acylations, N,N,N-trimethylation and O-carboxymethylation, which generate more soluble compounds with greater biocompatibility. For example, there are reports that such derivatives have been used to prepare nanoparticles for pulmonary delivery of various drugs for the treatment of cancer [155,156,157], tuberculosis [158], asthma [159], and COPD [160].

## 6. Patents

Over the past 10 years, several patents related to CS in pulmonary drug delivery devices have been reported (Table 2). The present review includes patents retrieved from Espacenet, an international patent database containing over 140 million patent documents. The search was performed using the following keyword combination: (“pulmonary drug delivery” or “pulmonary drug delivery”) and “chitosan nanoparticles”. It returned 151 patents, which were read to verify the use of CS nanoparticles for PDDS in local treatments. After that, only 11 patents were suitable for the purpose of the review. Among them, 63.64% were from the United States, 18.18% from China, and 9.09% from Australia and Turkey.

The objective of the patents included the development of new nanostructured carriers in which CS is an important component. Among the composition diversity, there are many nanotechnology structures, like solid lipid nanoparticles [161,162,163,164], liposomes [163,165], polymeric [163,164,166,167,168], magnetic [169], and inorganic nanoparticles [163]. The composition of nanoparticles varies in the chemical composition beyond chitosan, including phospholipid [161,162,165,166,167], cholesterol [161,162,165], biocompatible polymer [161,165,167,170,171], inorganic [161,163], sodium tripolyphosphate [166,167], cyclodextrin [166,167], chitosan derivatives [166,167], alginate [168], polyethylene glycol [168,171], fatty acid [162,171], iron [169], chromium [169], lipid [163,164,170,171], polymer [163,164,171], carbohydrate [170], polysaccharide [171], proteoglycan [171], glycosaminoglycan [171], and dendrimer [171]. In this sense, CS has many functions in these structures, showing its diversity of properties, such as increasing drug retention in lung tissue [165,168], and enhancing stability and bioavailability [167], surface functionalization [163,169,170], and permeation [171].

**Table 2:** Chitosan pulmonary drug delivery patents.

Patent Name	Patent Number	Country	Type	Chitosan Function	Disease	Active Pharmaceutical Ingredient Type	Ref.
Gsk3 inhibitor-loaded nano formulations as a cancer immunotherapeutic	WO2022006083A1	US	Lipid-based	Drug carrier	Cancer	GSK3 inhibitor	[161]
Novel method for dry powder inhalation comprising.	AU2014204483A1	AU	Lipidic	Enhance retention in lung tissue	Lung tissues diseases	not specified	[165]
Quercetin and paclitaxel co-transportation pulmonary-inhaled nanometer-targeted porous polymer particle and preparation method thereof	CN106309411A	CN	Polymeric	Formulation Ingredient	Lung cancer	Quercetin and paclitaxel	[166]
A pulmonary-inhaled chitosan-based nano-targeting polymer particles and its production method thereof	CN106265607A	CN	Polymeric	Enhancer of bioavailability and stability	Cancer	Monoclonal antibody cetuximab	[167]
Nano-delivery system for inhaled chemotherapy	WO2022119528A1	TR	Polymeric	Enhance retention in lung tissue	Lung cancer	Doxorubicin	[168]
Method of use for Apoe peptides	WO2023288316A1	US	Lipid-based	Targeting	Miscellaneous	Organic molecules, nucleic acid, peptides, and protein	[162]
Therapeutic methods and compositions comprising magnetizable nanoparticles	WO2022187556A1	US	Magnetic	Surface functionalization	Miscellaneous	Peptides, polymers, contrasting agents, imaging agents, and combinations thereof	[169]
Immunotherapeutic constructs and methods of their use	WO2021011496A1	US	Lipid-based, polymeric, and inorganic	Surface functionalization	Cancer	Antibody, nucleic acid, oligonucleotides, and small molecules	[163]
Hollow particles encapsulating a biological gas and methods of use	WO2014143808A1	US	Polymeric	Surface functionalization	Local or systemic hypoxia	Therapeutic gasses	[170]
Npc1 monobodies and monobody conjugates thereof	WO2022103840A2	US	Polymeric or lipid-based	Formulation Ingredient	Niemann-Pick disease	Peptides	[164]
Pd-l1-binding peptides and peptide complexes and methods of use thereof	WO2022115719A1	US	Not specified	Permeation enhancer	Cancer	Peptides	[171]

The patents have been developed to achieve delivery of many drugs such as quercetin [165], paclitaxel [165], antibodies [163,166], doxorubicin [167], nucleic acids [162], peptides [162,164,171], proteins [162], contrast agents [169], oligonucleotides [163], and therapeutic gases [170].

In addition to patents, the global chitosan market has significant growth potential, from USD 2.1 billion in 2022 to up to USD 8.5 billion in 2030. The pharmaceutical sector is the second fastest growing segment of the chitosan market. As previously described, Bioavanta-Bosti launched Novochizol™ in 2022. It is an aerosol based on chitosan nanoparticles that can be used to encapsulate small or large molecules for the treatment of COVID-19 [172,173].

## 7. Conclusions and Future Directions

Due to the increasing number of deaths from respiratory pathologies such as COVID-19, tuberculosis, and lung cancer, the development of innovative systems for pulmonary drug delivery has gained prominence in the scientific community. In this context, the use of nanostructured carriers has been widely studied, and among them, CS nanoparticles are emerging as a promising alternative. Indeed, the advancement of pulmonary drug delivery using CS is making significant progress, with notable achievements being observed to date.

CS is widely recognized as one of the most abundant renewable sources, second only to cellulose. In addition, it is characterized as a non-toxic, biocompatible and biodegradable polymer with a competitive advantage over other biodegradable polymers due to its inherent properties such as mucoadhesive, anti-inflammatory, and antimicrobial activities. In particular, the antimicrobial activity of CS can be exploited to minimize resistance to antimicrobials, such as *P. aeruginosa* infections in CS. In turn, its anti-inflammatory property may be useful to reduce the inflammatory process in cases of severe acute respiratory syndrome. In this sense, this review critically examined the advancements of CS-based nanoparticles as an inhaled drug delivery system.

Given their ability to improve local drug delivery, minimize side effects, enhance therapeutic activity and prolong drug release, these nanoparticles have enormous potential for clinical use. Indeed, the results evaluated in this review are encouraging. In addition to the advantages mentioned above, CS nanoparticles administered via inhalation have made it possible to overcome the major challenge associated with drug clearance from the lungs. In this sense, several studies have shown an increase in drug deposition in the lungs in in vivo models. The authors argue that this may be related to the important mucoadhesive property of CS, which facilitates drug penetration through the mucus layer. In addition, some studies have demonstrated the ability of CS nanoparticles to optimize the biopharmaceutical parameters of drugs, especially their solubility, contributing to an increase in their bioavailability. Moreover, a reduction in systemic toxicity was reported in some of the studies reviewed. On the other hand, several studies have demonstrated the ability of CS nanoparticles to enhance the therapeutic activity of drugs, especially in terms of increasing the antibacterial activity against CF-related *P. aeruginosa, Klebsiella pneumoniae*, and *M. tuberculosis*, the antiviral activity against SARS-CoV-2 and the anti-inflammatory activity required for the treatment of COPD, IPF and CF. Taken together, the observed benefits elevate chitosan to the status of a polymer with the most promising properties for the development of nanocarriers for pulmonary drug delivery applications.

However, further studies are needed to establish scalable processes for the preparation of CS nanoparticles. Furthermore, the surface engineering of CS nanoparticles through the use of specific ligands should be further explored to actively target these particles to specific sites in the lung. Also, the pharmacokinetic, preclinical toxicity, and biodistribution parameters of CS nanoparticles need to be studied in depth in order to move one step closer to conduct clinical trials.

## Figures and Tables

**Table 1:** CS-based nanoparticles for pulmonary drug delivery.

Disease	Drug	Limitations	Carrier	Main Results	Ref.
Asthma	Ferulic Acid	Low bioavailability and short half-life	Hyaluronic acid-coated CS NP	Improved drug interaction and transport across the mucus layer; increased therapeutic efficacy	[18]
	Budesonide	Low bioavailability	CS-coated PLGA NP	Improved bioavailability and in vivo lung deposition in animal model	[19]
	Baicalein	Low bioavailability	CS NP	Nanoparticles control the immune-allergy-inflammatory response of asthma in mice	[119]
	Montelukast	Significant hepatic metabolism after oral administration	CS NP	DPI formulation showed Optimum deposition in the deep lung	[120]
COPD	Budesonide	Low aqueous solubility and bioavailability	CS NP	Enhancement of drug solubility	[121]
	Amikacin	Poor lung penetration after endovenous administration	PEG-CS NP combined with black phosphorus quantum dots	Improved mucus penetration and antibacterial activity	[122]
Pulmonary fibrosis	Nifedipine	Low bioavailability	CS-PLGA NP	Reduced markers of pulmonary fibrosis and oxidative stress	[123]
IPF	msFGFR2c	Low bioavailability	Phosphoryl- choline-CS NP	Enhanced antifibrotic efficacy, reduced inflammatory cytokines, decreased pulmonary fibrosis score and collagen deposition	[124]
CF	Ciprofloxacin	Microbial resistance	ALG-lyase-functionalized CS NP	Higher inhibitory effect on *P. aeruginosa* biofilm	[20]
	wtCFTR-mRNA	Low stability; low transfection efficiency	CS-lecithin oil-core nanocapsules	Restored CFTR function in the cystic fibrosis cell line	[125]
	Antisense oligonucleotide (ASO)	Low stability	CS/ASO nanocomplex	Significant downregulation of ENaC activity in human respiratory epithelial cells	[126]
	Tobramycin	High frequency of administration; ototoxic and nephrotoxic effects; bacterial resistance	SLPICS-functionalized ALG/CS NP	Inhibition of *P. aeruginosa* in vitro; reduction in inflammatory response; improvement in interaction with CF mucus	[22]
	Ciprofloxacin	Microbial resistance	DNase-I-functionalized CS NP	Prolonged microbial inhibition, prevention of biofilm formation and biofilm dispersal potential	[127]
Lung cancer	Resveratrol	Low solubility	CS/lecithin nanocomplex	Enhanced antitumor activity; increased selectivity in A549 cells	[22]
	aPD-L1	Low stability; unwanted adverse effects	CS/aPD-L1 nanocomplex	Improved lung adhesion and permeation; enhanced therapeutic efficacy	[128]
Tuberculosis	Bedaquiline	Prolonged treatment; unwanted adverse effects	CS NP	Reduction in toxic effects; Increased drug concentration in the lungs	[15]
	Linezolid	Unwanted adverse effects	CS NPs	Improved deep lung deposition in vitro	[129]
Pneumonia	Gallium [Ga(III)]	Nephrotoxicity	Hyaluronic acid-CS NP	Improvement in Ga(III) persistence in the lungs and preventing its accumulation in the kidney	[130]
	Gentamicin	Low bioavailability; unwanted adverse effects	CS/Fucoidan NP	Improved antibacterial activity; reduced systemic toxicity	[131]
RSV	Oxymatrine	Enzymatic degradation; poor lung penetration	CS-coated liposomes	Enhanced distribution and retention of oxymatrine in lung tissue in vivo	[132]
COVID-19	Silymarin and curcumin	Low penetration and adsorption in the lungs	CS-coated BSA NP	Reduced inflammation; enhanced antiviral activity in vitro	[133]

ALG: alginate; aPD-L1: anti-programmed cell death protein ligand 1; ASO: antisense oligonucleotide; BSA: bovine serum albumin; CF: cystic fibrosis; CFTR: cystic fibrosis transmembrane conductance regulator; COPD: chronic obstructive pulmonary disease; CS NPs: chitosan nanoparticles; DPI: dry powder inhaler; ENaC: epithelial sodium channel; IPF: idiopathic pulmonary fibrosis; PEG: polyethylene glycol; PLGA: poly(lactic-co-glycolic acid); msFGFR2c: S252 W mutant soluble ectodomain of fibroblast growth factor receptor-2 IIIc; SLPICS: secretory leukocyte protease inhibitor; RSV: Human respiratory syncytial virus.

## Data Availability

Not applicable.
